# Anxiety and Social Support as Predictors of Student Academic Motivation During the COVID-19

**DOI:** 10.3389/fpsyg.2021.644338

**Published:** 2021-05-24

**Authors:** Ana Camacho, Nadine Correia, Sonia Zaccoletti, João R. Daniel

**Affiliations:** ^1^Faculty of Psychology and Education Sciences, University of Porto, Porto, Portugal; ^2^School of Health, Polytechnic of Porto, Porto, Portugal; ^3^Instituto Universitário de Lisboa (ISCTE-IUL), CIS-IUL, Lisbon, Portugal; ^4^Department of Developmental and Socialization Psychology, University of Padova, Padua, Italy; ^5^William James Center for Research, ISPA–Instituto Universitário, Lisbon, Portugal

**Keywords:** COVID-19, pandemic, students, academic motivation, anxiety, social support, remote learning, parents’ perceptions

## Abstract

In this study we examined whether parents’ perceptions of students’ anxiety as well as perceived support from both teachers and classmates were predictive of changes in students’ academic motivation during the first wave of COVID-19. To this end, we used a retrospective pretest-posttest design together with a latent change score model to analyze our data. From April to May of 2020, 394 Portuguese parents of students in grades 1–9 participated in this study. Our results showed that students’ anxiety and teachers’ social support, as perceived by parents, were highly significant predictors of academic motivation changes. Specifically, we found a negative effect of anxiety and a positive effect of teachers’ social support on students’ academic motivation. Our results did not show, however, a significant predictive role of classmates’ social support. This study provides an important contribution to further understand the intrapersonal and interpersonal factors that are associated with the decline of students’ academic motivation during the COVID-19 pandemic. The pivotal role of teachers in sustaining students’ academic motivation and other relevant educational implications for the ongoing pandemic are discussed.

## Introduction

The disruptive effects of the COVID-19 pandemic have profoundly impacted all sectors of society, including education. Home confinement measures, school closures, and a sudden shift to remote learning imposed substantial changes to teachers, students, and their families’ daily lives. According to a United Nations policy brief of August 2020, nearly 1.6 billion students in more than 190 countries from all continents were affected by the COVID-19 (United Nations, 2020).

Parents had to provide close support to their children, acting as home tutors. Teachers had to switch from traditional face-to-face classes to alternative forms of distance education, not only embracing new methods, but also ensuring close support for students and their parents. On top of facing a global health emergency that generates fear and anxiety, students shifted to online learning, which required quick adjustments and affected their daily habits, experiences, and expectations. These changes may have required more self-motivation to learn, in a situation characterized by potential less direct support from teachers and classmates (Aucejo et al., 2020).

A recent study, examining the impact of COVID-19 restrictions, has reported a decline in students’ academic motivation both in Portugal and in Italy (Zaccoletti et al., 2020). Yet, little is known about the intrapersonal and interpersonal factors that are associated with this decline. As such, it is important that both researchers and practitioners further study the impact of COVID-19 pandemic on students to find ways to mitigate its negative consequences.

In the present study, we aimed to examine the predictive role of anxiety and social support, from teachers and classmates, in the changes of Portuguese students’ academic motivation, as perceived by parents. For this purpose, we surveyed 394 parents by means of an online survey during April and May of 2020. We sampled parents—rather than their children—due to time constraints and ethical reasons surrounding the participation on an online survey.

In the following sections, we will address: (a) research on student academic motivation; (b) the impact of COVID-19 on students’ anxiety; (c) the role of perceived social support for students; and (d) an overview of the Portuguese educational context.

### Academic Motivation

The study of achievement motivation has a long tradition in Educational Psychology (Murphy and Alexander, 2000; Wigfield and Koenka, 2020). Also, in Cognitive Psychology, since the 2000s researchers have focused on the interplay between motivation and cognition, thus acknowledging that motivational states influence cognitive processing (Markman et al., 2005).

Achievement motivation—also coined as competence motivation—can be defined as the “energization and direction of behavior with regard to effectiveness, ability, sufficiency, or success” (Elliot et al., 2017). A large body of meta-analyses and empirical studies have shown that motivation is a medium to strong predictor of academic achievement (e.g., Guay et al., 2010; Cerasoli et al., 2014; Taylor et al., 2014; Kriegbaum et al., 2018). Moreover, motivation was found to contribute to academic achievement even when cognitive skills were jointly considered (Wigfield and Wentzel, 2007). In light of these findings, researchers designed motivational interventions to enhance students’ academic achievement. Overall, these interventions were effective in promoting achievement outcomes in diverse domains such as overall GPA, reading, writing, science, and maths (e.g., Wigfield and Wentzel, 2007; Lazowski and Hulleman, 2016; Camacho et al., 2020). Altogether, prior research underlined the pivotal role of motivation in the school context and the promising effects of motivation interventions.

From a theoretical standpoint, several motivation-related theories have been proposed (e.g., self-determination theory, expectancy-value theory, social cognitive theory, self-theories). Despite the differences, these theories share communalities. One communality is the importance attributed to the social context in shaping students’ motivation. Parents can facilitate or undermine the development of motivational resources in their children through their socialization practices (Grolnick et al., 2009). In the same line, school settings directly or indirectly influence students’ motivation (Anderman and Gray, 2017) and classmates influence students’ motivation and engagement (Ladd et al., 2009). Interestingly, perceived teacher social support was found to remain a significant predictor of academic motivation even when perceived support from parents and classmates were considered (Wentzel, 2009).

Despite communalities between motivation theories, the self-determination theory seems a particularly useful theory to frame empirical research on academic motivation during the COVID-19. According to the self-determination theory, students’ intrinsic motivation depends on the satisfaction of basic psychological needs for competence, autonomy, and relatedness (Wentzel, 2009). Competence refers to the need to perceive mastery in one’s pursuits and interactions with the social environment; autonomy refers to the perception of psychological freedom and being a causal agent of one’s own life; and relatedness refers to the importance of establishing emotional bonds and being in interaction with other people. Students fulfill these psychological needs and consequently become more intrinsically motivated when teachers and classmates provide authentic, warm and supportive environments (Reeve, 2002; Ryan and Deci, 2020). However, the home confinement and the shift to distance learning methods adopted during the first wave of the COVID-19 may have threatened students’ fulfillment of the three basic psychological needs, thereby hindering students’ intrinsic motivation for school (Zaccoletti et al., 2020). Recent studies showed indeed a decline in students’ academic motivation during the COVID-19 pandemic, with younger students showing a greater decrease in their motivation (Pasion et al., 2020; Zaccoletti et al., 2020). Nevertheless, while examining the role of demographic variables in the trajectory of academic motivation during the COVID-19 pandemic is relevant, understanding the predictive role of intrapersonal and interpersonal variables—such as anxiety and perceived social support—is also important to further understand the impact of COVID-19 on students.

### COVID-19 and Students’ Anxiety

The COVID-19 outbreak imposed school closures worldwide. Students were forced to move to online learning, with no prediction of returning to face-to-face classes. This emergency situation and the sudden need to change habits and routines (Duan and Zhu, 2020) impacted students’ perceptions of safety and preparedness to adapt to new learning methods, thereby leading to increased levels of anxiety and stress (Unger and Meiran, 2020).

Anxiety can be defined as a subjective state of fear and apprehension, thus causing physiological arousal such as rapid heart rate, hyperventilation, and sweating (Eysenck, 1992). Worry and concern refer to the cognitive component of anxiety such as intrusive thoughts and perception of vulnerability (Putwain, 2007). Stress was originally conceived as a state of adaptation to environmental pressures (Selye, 1956), which can have either positive or negative outcomes (Putwain, 2007). Despite the different meanings, researchers sometimes use these constructs interchangeably to refer to a state of unpleasant emotional state (Putwain, 2007).

Anxiety, stress and other unpleasant emotional states are common psychological responses to catastrophes or emergencies, such as public health emergencies (Rubin and Wessely, 2020). Moreover, these events can be traumatic, leading to a sense of insecurity and triggering anxiety disorders, such as post-traumatic stress disorder. Nonetheless, the characteristics of a catastrophe and an epidemic outbreak are distinct. In an epidemic outbreak such as COVID-19, contrary to what happens in a catastrophe, location, scope, and duration are uncertain, which is more likely to contribute to imbalance and lack of sense of security and control (Li et al., 2020).

Recent research has identified distressing psychological consequences related with the COVID-19 pandemic, such as worry, fear, and anxiety (Cao et al., 2020; Chen et al., 2020). Research has also suggested that, in addition to remote learning, isolation and lack of social contact during the pandemic may have led to an increased sense of fear, stress, anxiety, and even depression (Hiremath et al., 2020).

Overall, students’ anxiety has been negatively linked to their academic motivation (Omidvar et al., 2013). Also, the shift to remote and online distance learning has been described as possibly compromising students’ motivation (Breneiser et al., 2018). Despite the importance of this evidence, there is limited research on the associations between students’ anxiety and their academic motivation during the COVID-19.

### The Role of Perceived Social Support for Students

Due to its complexity, academic motivation is influenced not only by intrapersonal factors, but also by the broader social context by which students are surrounded. In fact, students’ academic attitudes and behaviors are strongly influenced by key social agents, such as teachers, parents, classmates, and friends (Legault et al., 2006). The positive role of social support in academic motivation has been documented (e.g., Tezci et al., 2015), with prior research suggesting positive associations between students’ academic motivation and support received from their parents, teachers, and friends (e.g., Atnafu, 2012; High and Scharp, 2015; Jiang et al., 2015).

Social support can be defined as “the processes of social exchange that contribute to the development of individuals’ behavioral patterns, social cognitions, and values” (Farmer and Farmer, 1996, p. 433). It is also described as promoting the motivation needed to achieve success, and to cope effectively with stressful events (Tezci et al., 2015).

The role that significant others may play, and how their support may influence students, can be interpreted considering the cognitive evaluation theory, under the umbrella of self-determination theory (Deci and Ryan, 1985, 2002). According to the cognitive evaluation theory, constructive interpersonal support promotes self-determined motivation. In other words, social contexts and key social agents are crucial to fulfill students’ basic psychological needs for autonomy, competence, and relatedness, which will facilitate intrinsic and internalized motivation (Deci and Ryan, 1985, 2002).

Research suggests, for instance, that students’ motivation benefits when teachers support their *autonomy* (e.g., Reeve, 2002). Existing studies equally point out that constructive feedback and information exchange between students and their teachers, parents, classmates, and friends may fulfill their *competence* needs (Ryan et al., 1994). *Relatedness* has also been shown to have a powerful effect on academic motivation (Furrer and Skinner, 2003), and the role of social support in academic motivation has been well established in self-determination theory research. Further, support from teachers, parents, classmates, and friends is described as having a cumulative effect (Green-Demers, 2006).

The role of social support in coping with adversity and emergencies more effectively has also been extensively reported (e.g., Masten, 2001). The establishment of relationships with teachers, family, classmates, friends, and other significant adults characterized by emotional and practical support build students’ resilience (Taylor et al., 2010).

Particularly during the pandemic, as physical isolation measures were implemented (e.g., social distancing, home confinement), several social support networks may have been suspended (Taylor et al., 2010). Previous studies have already suggested that social support, as perceived by students, was negatively associated with students’ anxiety during the pandemic (Cao et al., 2020; Chen et al., 2020; Ma and Miller, 2020). Importantly, perceiving the existence and availability of sources of social support may have contributed to better cope with anxiety related to COVID-19 (Ma and Miller, 2020).

These findings underline the importance of social support to safeguard both students’ academic motivation and psychological health. However, to our knowledge, no study has examined how social support was associated with students’ academic motivation specifically during the first wave of the COVID-19 pandemic, while simultaneously considering students’ anxiety.

### Portuguese Educational Context

The Portuguese education system comprises 12 years of compulsory education, divided into basic education (9 years) and secondary education (3 years). In this study we will focus on students attending basic education, which is divided into three cycles: first cycle (i.e., grades 1–4), second cycle (i.e., grades 5 and 6), and third cycle (i.e., grades 7–9) (EACEA/EURYDICE, 2019).

Similar to what happened around the world, the Portuguese government decreed home confinement during the first wave of COVID-19. This measure implied the closure of schools nationwide from 16th March until the end of the school year (Decree-Law no. 14/2020, 2020). Of note, some families self-isolated on their own initiative since the beginning of March.

Following school closures, teachers had to adopt new strategies to ensure that students had access to instruction, even if remotely. Distance, online learning approaches were therefore privileged, implying an ongoing adaptation process on the part of all students, parents, and teachers. This process uncovered the existence of inequalities in the country (e.g., access to electronic equipment, maintenance of individualized support), which prompted several responses from both central government and local institutions. An example refers to broadcasting educational content on national television (Flores and Gago, 2020). Due to these measures, parental and teacher support became indispensable to support students’ academic motivation. Nonetheless, as suggested by recent research, Portuguese students experienced a decrease in their academic motivation with the onset of COVID-19 (Zaccoletti et al., 2020).

## The Present Study

Although much research has been conducted on the impact of COVID-19 on the daily lives of students, there are noteworthy research gaps that warrant further empirical enquiry. First, there is still little research on students’ academic motivation during the COVID-19 (Zaccoletti et al., 2020). This is a noteworthy gap since motivation is a strong predictor of key academic skills and ultimately contributes to students’ psychological well-being, academic achievement, and school completion (e.g., Guay et al., 2008; Archambault et al., 2009; Lai, 2011; Cerasoli et al., 2014; Kriegbaum et al., 2015; Lazowski and Hulleman, 2016; Zaccoletti et al., 2020). Second, as far as we know, no study to date inspected the predictive role of students’ anxiety and perceived social support to changes in academic motivation during the COVID-19 pandemic. Third, a recent systematic review underlined that few studies focused on parents’ views on the psychological, educational, academic, physical, and emotional impact of the first home confinement period on students (Cachón-Zagalaz et al., 2020).

Therefore, we addressed these research gaps in the present study. Using parents as informants, we tested whether anxiety and perceived social support from teachers and from classmates were predictive of changes in students’ academic motivation during the first wave of the COVID-19. We formulated three hypotheses: We anticipated that more anxious students would experience a greater decrease in their academic motivation (H1); We hypothesized that higher social support from teachers would be associated with a lower decrease in students’ academic motivation (H2); Similarly, we expected that higher social support from classmates would be associated with a lower decrease in students’ academic motivation (H3).

To accomplish these aims, we surveyed 394 Portuguese parents of children in grades 1–9, who completed an online survey during the first wave of COVID-19. We enrolled parents in our study—rather than their children—due to four reasons. First, the participation of young children in online surveys raises ethical and safety concerns. Second, children as young as 6-years old (i.e., first graders) could not ascertain about their academic motivation, anxiety and perceived social support since they are still learning how to read and write. Third, previous studies have shown that parents are reliable sources of information concerning their children’s emotions and behaviors (e.g., Gilger, 1992; Allerhand, 2020; Owens et al., 2020; Saçkes et al., 2016). Fourth, a recent systematic review stressed the need for more research focusing on parents’ views, who spent a considerable amount of time with their children during the home confinement period (Cachón-Zagalaz et al., 2020).

## Materials and Methods

### Participants

Three hundred ninety-four Portuguese parents of students in grades 1–9 participated in our study (see Table 1 for detailed demographic information). Each parent was asked to bear in mind only one child while answering the survey, even if they had more than one eligible child. This sample is part of a larger cross-country sample previously used in Zaccoletti et al.’s (2020) study.

**TABLE 1:** Participants’ sociodemographic characteristics.

***Parents* (*N* = 394)**	
Mother	*n* = 365 (92.4%)
Father	*n* = 29 (7.3%)
Age	*M* = 41 (*SD* = 5.53)
Educational level in years	*M* = 15 (*SD* = 6.23)
Professional situation affected by the pandemic	
Yes	*n* = 172 (43.7%)
No	*n* = 222 (56.3%)
Students	
Female	*n* = 191 (48.5%)
Male	*n* = 203 (51.5%)
Age	*M* = 10.04 (*SD* = 2.52)
Grade-level	
Grades 1–4	197 (50%)
Grades 5–6	105 (34%)
Grades 7–9	92 (16%)

Parents’ average age was 41 (*SD* = 5.53). Parents’ gender was unevenly distributed as we received mostly answers from mothers (*n* = 365). Regarding the educational level, parents reported 15 average years of instruction (school plus higher education). Concerning their work, 172 parents referred that the pandemic affected their professional situation somehow (e.g., unemployment, lay-off, remote work).

Students’ average age was 10.04 (*SD* = 2.52). Students’ gender distribution was balanced (*n*_*girls*_ = 191, *n*_*boys*_ = 203). As for the grade-level, 50% of the students were in grades 1–4, 34% were in grades 5–6 and 16% were in grades 7–9.

### Instruments

#### Students’ Academic Motivation

We used a set of items from the standardized battery AMOS 8–15 (Cornoldi et al., 2005) to assess students’ academic motivation. The motivational scale comprised five items, which were scored on a five-point Likert scale, ranging from 1 (I completely disagree) to 5 (I completely agree) (for further details, see Zaccoletti et al., 2020). Two examples of items were: “When the teacher assigns homework, my child does it by self-initiative and not because the parents ask her/him to” and “My child studies the minimum to get a sufficient grade” (reversed item). Higher scores indicated higher intrinsic motivation for school tasks. We used a retrospective pretest–posttest design (Little et al., 2020), thus asking parents to report their perceptions of children’s academic motivation in two timepoints: (1) before the onset of COVID-19; (2) during the first wave of the COVID-19. Items were highly reliable at both timepoints (McDonald’s ωt_*preCOVID–*__19_ = 0.88; McDonald’s ωt_*COVID–*__19_ = 0.89).

#### Students’ Anxiety

We asked parents a single question to measure their perception of children’s anxiety during the first wave of COVID-19: “Over the last month, to what extent do you consider that your child felt anxious due to the COVID-19 pandemic?” Parents rated the single item on a 5-point Likert scale, ranging from 1 (Never) to 5 (Always).

#### Students’ Social Support

We used two separate items to assess parents’ perceptions of social support provided by teachers and classmates to their children: “Do you think teachers are a source of social support to your child during this period of social isolation?” and “Do you think classmates are a source of social support to your child during this period of social isolation?” Parents rated both items on a 5-point Likert scale, ranging from 1 (Completely false) to 5 (Completely true).

### Data Collection

Data collection occurred in April and May of 2020, during the first wave of the COVID-19 pandemic in Portugal. We developed an online survey using Qualtrics XM Platform (Qualtrics, 2019 Provo, UT). The survey was then disseminated to Portuguese parents through: (a) official university channels (i.e., university website, newsletters, and emails); and (b) social media networks (i.e., LinkedIn and Facebook groups). Parents were provided with a detailed consent letter in compliance with the General Data Protection Regulation. Only parents who consented to participate—by checking a box on the online survey—were enrolled in our study. Although we used convenience sampling, we ensured that parents from all Portuguese districts were represented in our sample.

## Results

### Data Analysis Plan

We used a latent change score model (LCSM; Kievit et al., 2018) to test whether parents’ perceptions of students’ anxiety, teachers’ social support and classmates’ social support were predictive of motivation changes, while controlling for the effects of children’s age and gender. LCSMs are a class of Structural Equation Models (SEM) that allow testing a wide range of hypotheses about a psychological variable of interest, measured at two time points. LCSM four parameters of interest are the: (1) pre-COVID mean latent motivation score; (2) mean latent change score (i.e., the rate of change in motivation); (3) latent change score variance (i.e., individual variation in the rate of change in motivation); and the (4) covariance between pre-COVID-19 motivation and the mean latent change score (i.e., the dependence of rate of change on initial motivation scores). All measurement model parameters were constrained to be equal across time (please see Zaccoletti et al., 2020 for further details on the invariance tests). Most relevant for this study is the estimate of the mean latent change score and the estimates of the regression paths linking the predictor variables to this latent score. All predictive variables were grand-mean centered before being included in the model. As such, the motivation change latent score (a latent intercept in SEM terminology), estimated by the LCSM, corresponds to the estimated change in motivation for the average student. All LCSMs were estimated using the lavaan package (version 0.6-5; Rosseel, 2012) in R (version 3.6.1; R Core Team, 2019).

### Descriptive Statistics

Table 2 summarizes the descriptive statistics (means, standard deviations, and correlation coefficients) for academic motivation, anxiety, and social support. Correlational analysis showed that (1) all motivation items were negatively and significantly correlated with students’ anxiety, with the correlation magnitudes being higher for the COVID-19 period (correlations range: *r*s_*pre–COVID–*__19_ = −0.12 to −0.15; *r*s_*COVID–*__19_ = −0.13 to −0.24); (2) except for one item, all other motivation items were positively and significantly correlated with teachers’ social support, with the correlation magnitudes, again, being higher for the COVID-19 period (correlations range: *r*s_*pre–COVID–*__19_ = 0.09 to 0.15; *r*s_*COVID–*__19_ = 0.14 to 0.20); and (3) only three motivation items, referring to the COVID-19 period, were positively and significantly correlated with classmates’ social support, with the correlation magnitudes being lower than for anxiety and teachers’ social support.

**TABLE 2:** Correlation matrix with Means (*M*) and Standard Deviations (*SD*).

***N* = 394**	***M***	***SD***	**1**	**2**	**3**	**4**	**5**	**6**	**7**	**8**	**9**	**10**	**11**	**12**	**13**	**14**
**Motivation**																
*Pre-COVID-19*																
M1	3.10	1.24														
M2	3.28	1.22	0.58**													
M3	3.19	1.19	0.61**	0.75**												
M4	3.46	1.23	0.44**	0.54**	0.58**											
M5	3.42	1.15	0.50**	0.69**	0.61**	0.49**										
*COVID-19*																
M1	3.01	1.25	0.41**	0.48**	0.46**	0.24**	0.42**									
M2	3.21	1.18	0.43**	0.65**	0.60**	0.35**	0.54**	0.73**								
M3	2.99	1.15	0.42**	0.55**	0.66**	0.34**	0.50**	0.70**	0.79**							
M4	3.24	1.17	0.37**	0.46**	0.50**	0.70**	0.41**	0.48**	0.56**	0.58**						
M5	3.26	1.16	0.39**	0.55**	0.47**	0.35**	0.68**	0.57**	0.67**	0.62**	0.48**					
**Predictors**																
Anxiety	2.88	0.94	−0.12*	−0.13*	−0.15**	−0.10*	−0.12*	−0.24**	−0.20**	−0.23**	−0.20**	−0.13**				
Teachers’ social support	3.57	1.18	0.15**	0.13*	0.09	0.14**	0.11*	0.20**	0.18**	0.16**	0.19**	0.14**	−0.00			
Classmates’ social support	3.64	1.19	0.04	0.08	0.06	0.05	0.04	0.08	0.12*	0.08	0.11*	0.12*	0.04	0.41**		
Age	10.04	2.52	0.00	0.04	0.11*	−0.10*	0.08	0.09	0.18**	0.20**	0.02	0.18**	0.01	−0.07	0.14**	
Gender	0.52	0.50	0.18**	0.14**	0.11*	0.23**	0.13**	0.09	0.12*	0.13**	0.20**	0.16**	0.04	0.08	−0.10	0.14**

Gender mean values represent the proportion of boys in the sample. Estimates of the LCSM presented in the next table can be reproduced using the correlation matrix *p < 0.05 and **p < 0.01.

### Latent Change Score Model

**FIGURE 1:**
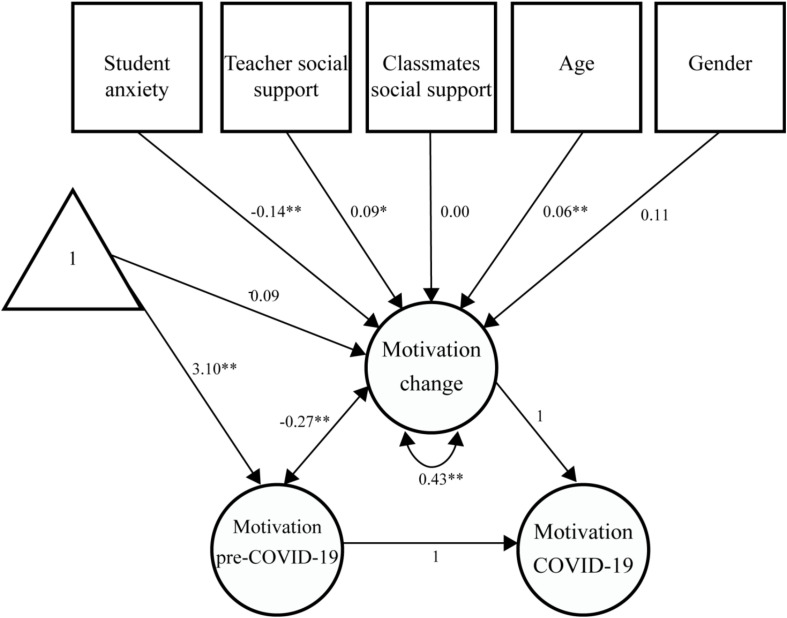
Unstandardized model estimates. For simplicity, only estimates of interest of the LCSM are presented. Shapes follow standard figural notation for structural equation modeling: triangle—intercepts (estimate mean levels), squares—manifest/observed variables, circles—latent variables, one-headed arrows—unidirectional effects (regression weights or means), and double-headed arrows—(co-) variances.

The aforementioned correlation patterns are captured in the Latent Change Score Model (LCSM) regression estimates (Figure 1; see Table 3 for complete model estimates). The LCSM estimated a conditional 0.09 non-significant (*p* = 0.172) drop in motivation for the average child (as perceived by their parents), following a pre-COVID-19 mean latent motivation score of 3.10. Higher drops in motivation were significantly associated with higher pre-COVID-19 scores (co-variance = −0.27, *p* < 0.001). Also, model estimates showed a significant individual variability in the rates of change (latent change score variance = 0.43, *p* < 0.001).

**TABLE 3:** Unstandardized latent change score model estimates.

	**Estimate**	***SE***	***p***
**1. Regression estimates (Motivation latent change)**			
Motivation change ∼ Students’ anxiety	–0.14	0.04	<0.001
Motivation change ∼ Teachers’ social support	0.09	0.03	0.006
Motivation change ∼ Classmates’ social support	0.00	0.04	0.957
Motivation change ∼ Age	0.06	0.01	<0.001
Motivation change ∼ Gender	0.11	0.07	0.120
**2. Factor loadings (Motivation items)**			
Motivation pre-COVID-19 = ∼ M1	1.00		
Motivation pre-COVID-19 = ∼ M2	1.14	0.05	<0.001
Motivation pre-COVID-19 = ∼ M3	1.08	0.05	<0.001
Motivation pre-COVID-19 = ∼ M4	0.83	0.06	<0.001
Motivation pre-COVID-19 = ∼ M5	0.89	0.05	<0.001
Motivation COVID-19 = ∼ M1	1.00		
Motivation COVID-19 = ∼ M2	1.14	0.05	<0.001
Motivation COVID-19 = ∼ M3	1.08	0.05	<0.001
Motivation COVID-19 = ∼ M4	0.83	0.06	<0.001
Motivation COVID-19 = ∼ M5	0.89	0.05	<0.001
**3.Intercepts (means)**			
**3.1. Motivation items**			
M1 pre-COVID-19	0.00		
M2 pre-COVID-19	–0.25	0.16	0.112
M3 pre-COVID-19	–0.17	0.15	0.279
M4 pre-COVID-19	0.89	0.20	<0.001
M5pre-COVID-19	0.67	0.17	<0.001
M1COVID-19	0.00		
M2 COVID-19	–0.21	0.15	<0.001
M3COVID-19	–0.27	0.15	<0.001
M4COVID-19	0.73	0.19	<0.001
M5COVID-19	0.59	0.16	<0.001
**3.2. Motivation latent variables**			
Motivation pre-COVID-19	3.10	0.62	<0.001
Motivation COVID-19	0.00		
Motivation change	–0.09	0.07	0.172
**4. Variances**			
**4.1. Motivation items**			
M1 pre-COVID-19	0.82	0.09	<0.001
M2 pre-COVID-19	0.34	0.05	<0.001
M3 pre-COVID-19	0.38	0.05	<0.001
M4 pre-COVID-19	0.91	0.08	< 0.001
M5 pre-COVID-19	0.58	0.06	<0.001
M1 COVID-19	0.58	0.07	<0.001
M2 COVID-19	0.25	0.04	<0.001
M3 COVID-19	0.30	0.04	<0.001
M4 COVID-19	0.80	0.07	<0.001
M5 COVID-19	0.63	0.05	<0.001
**4.2. Motivation latent change**			
Motivation pre-COVID-19	0.00		
Motivation COVID-19	0.86	0.07	<0.001
Motivation change	0.43	0.07	<0.001
**5. Co-variances**			
Motivation pre-COVID-19 ∼∼ Motivation change	–0.27	0.05	<0.001
**6.Correlated error terms**			
M1 pre-COVID-19 ∼∼ M1 COVID-19	0.08	0.04	<0.001
M2 pre-COVID-19 ∼∼ M2 COVID-19	0.08	0.03	<0.001
M3 pre-COVID-19 ∼∼ M3 COVID-19	0.15	0.03	<0.001
M4 pre-COVID-19 ∼∼ M4 COVID-19	0.60	0.06	<0.001
M5 pre-COVID-19 ∼∼ M5 COVID-19	0.30	0.04	<0.001

*“∼,” “= ∼,” and “∼∼” symbols follow lavaan R package (Rosseel, 2012) operators’ terminology and stand for “regressed on,” “is measured by,” and “correlated with,” respectively.*

Concerning our predictor variables, the LCSM also indicated that higher drops in motivation were associated with: (1) higher anxiety scores (*ß* = −0.14, *SE* = 0.04, *p* < 0.001); (2) lower teacher social support (*ß* = 0.09, *SE* = 0.03, *p* = 0.006); (3) and younger children (*ß* = 0.06, *SE* = 0.01, *p* < 0.001). Classmates’ social support and gender had no significant effect on motivation change (*p*s > 0.05; see Table 3 for regression estimates).

## Discussion

In this study, we intended to deepen results obtained in a previous study documenting a decrease in students’ academic motivation during the COVID-19, both in Italy and in Portugal (Zaccoletti et al., 2020). Specifically, this study aimed to examine the role of students’ anxiety and social support (i.e., teachers’ social support and classmates’ social support) as predictors of the decrease in students’ motivation, as perceived by parents. To that end, we surveyed 394 Portuguese parents of students in grades 1–9 using an online survey distributed from April until May of 2020 (i.e., during the first wave of the COVID-19).

Our results showed that students’ anxiety and teachers’ social support, as reported by parents, were significant predictors of the decrease in students’ academic motivation during this time. Regarding students’ anxiety, we found a negative association between anxiety and academic motivation. Our first hypothesis (H1) was thus confirmed, as more anxious students experienced greater decreases in their academic motivation, based on parents’ perceptions. This finding is in line with prior evidence reporting that psychological factors such as anxiety, stress, and grief during emergency situations and quarantines have detrimental effects on learning (Di Pietro et al., 2020). Recent studies examining the impact of COVID-19 on mental health indicators have already shown that students—from primary school to university—experienced a rise in psychological symptoms such as anxiety, stress, and depression (Alemany-Arrebola et al., 2020; Cachón-Zagalaz et al., 2020; Li et al., 2020; Rodríguez-Hidalgo et al., 2020). One of these studies further indicated that university students’ anxiety during COVID-19 was negatively related to their academic self-efficacy (Alemany-Arrebola et al., 2020), which is one dimension of academic motivation. In addition, our results concur with research indicating that students’ stress and anxiety are negatively associated with their academic motivation (Omidvar et al., 2013).

We also aimed to investigate the predictive role of social support on academic motivation. Based on parents’ reports, higher social support from teachers was associated with lower decrease in students’ academic motivation, thus confirming our second hypothesis (H2). This finding concurs with previous studies showing that perceived social support from teachers is positively linked to different dimensions of academic motivation (e.g., Wentzel, 1998, 2009; Wentzel et al., 2010; Song et al., 2015). During the first wave of COVID-19, teachers had to master significant challenges. Specifically, they had to adapt to new teaching formats, while maintaining close contact with students and their families, ensuring that students stayed engaged and did not lose their motivation (König et al., 2020). For this reason, during this unprecedented emergency situation, parents may have perceived and valued teachers’ crucial role in supporting students’ academic motivation.

This finding is consistent with evidence emphasizing that when teachers are involved, provide structure, and establish an autonomy-supportive environment, they contribute to fulfill students’ basic psychological needs of relatedness, competence, and autonomy (Deci and Ryan, 1985, 2002; Reeve, 2002; Wentzel, 2009). Basic psychological needs–which are critical to sustain students’ academic motivation–may have been especially compromised during the COVID-19 pandemic (Zaccoletti et al., 2020). The establishment of emotionally close and trusting relationships with teachers is a pathway to develop students’ academic motivation and well-being (Wentzel, 2009). Consequently, our findings also stress the importance of collaborative relationships between teachers, students, and parents, particularly during challenging times (Pajarianto et al., 2020).

Unexpectedly, our findings showed that higher classmates’ social support was not significantly associated with lower decrease in students’ academic motivation, according to parents’ perceptions. Therefore, our results failed to support our third hypothesis (H3). One possible explanation for this finding is that we relied on parents’ reports rather than on students themselves. Possibly, students could have perceived classmates as a more important source of social support than parents did. In fact, previous studies based on students’ perceptions have documented the prominence of classmates as sources of social support, or even similar importance attributed to classmates and teachers (Bokhorst et al., 2010). Specifically, empirical evidence suggests that students tend to rank teachers as most important for providing informational and instrumental support (Lempers and Clark-Lempers, 1992), and to rank classmates as most important for providing informational and emotional support (Reid et al., 1989; Hombrados-Mendieta et al., 2012).

Overall, prior research has demonstrated a positive link between multiple sources of social support and students’ behavioral, emotional, and academic adjustment (e.g., Cook et al., 2002). Research has particularly suggested the importance of both teachers and classmates as sources of social support for students (Eccles and Roeser, 2003).

Although our study showed a negative effect of anxiety and a positive effect of teachers’ social support on students’ academic motivation, our results need to be interpreted with caution since we relied on parents’ perceptions. Studies examining the impact of anxiety and sources of social support on academic motivation during the COVID-19 that rely directly on students’ perceptions are highly needed.

### Limitations and Future Research

This study has some limitations that could stimulate future research. First, data was collected using a convenience sampling method, therefore our sample is not representative of the Portuguese population. Nevertheless, our sample included parents from all Portuguese districts.

Second, we used a retrospective pretest-posttest design (Little et al., 2020), which requires some prudence in making sense of students’ academic motivation trajectory, before and after the COVID-19. Particularly, insufficient recall or negatively biased responses due to the unpredictability and constraining situation created by COVID-19 lockdowns might have occurred. Future research, using datasets dating back to pre-COVID-19, might help researchers further explore this trajectory.

Third, in this study we relied on parents as informants, using an online survey and considering one single level of analysis (i.e., parents’ perceptions). In effect, most studies conducted during COVID-19 have used online surveys and self-assessment scales (Saravanan et al., 2020). Also, in relation to the survey, we used single items to operationalize two of our explanatory variables. Although this choice raises concerns, there are several empirical studies, across a range of fields, supporting the use of single items in some cases. For example, for practical reasons (e.g., reduce the length of a survey to avoid more desirable response rates and decrease non-completion rates), or due to a higher predictive power of single items vs. multiple-item scales (e.g., Bergkvist and Rossiter, 2007; Hoeppner et al., 2011; Ahmad et al., 2014; Fisher et al., 2016; Williams and Smith, 2016). Nonetheless, future research might consider multiple informants (e.g., teachers, students), different levels of analysis (e.g., teachers’ practices, students’ strategies) and complementary methods (e.g., interviews) to achieve a comprehensive understanding of students’ academic motivation, its changes, and determinants. Additional research enrolling school-aged samples is highly needed as current empirical publications relating COVID-19 with education are mostly focused on university students. In the same line, studying academic motivation, anxiety, and perceived social support of vulnerable student populations (e.g., students with special needs and students from disadvantaged backgrounds) would be an important research endeavor.

Fourth, we acknowledge that the age span of our sample is large. However, sample size was not sufficient to break it into smaller samples, with robust sample sizes that would allow further analysis of developmental differences.

Fifth, we did not consider students’ academic achievement or other psychological outcomes besides students’ motivation (e.g., self-regulation), which may be addressed in future research.

Finally, our data was collected in April and May 2020. Thus, we may hypothesize that the magnitude of the association found could differ if data had been collected in March, when face-to-face learning was suspended. In effect, at an earlier stage of the spread of COVID-19 and of the implementation of restrictive measures, students may have been more anxious and psychologically distressed (Saravanan et al., 2020).

### Educational Implications

COVID-19 has dramatically changed the daily routines of students, teachers, and parents, who faced increased anxiety and had to adapt to new learning methods. Although our study followed a correlational research design, some educational implications may be discussed. First, both parents and teachers need to be aware and to monitor students’ anxiety since it was negatively associated with academic motivation during the first wave of COVID-19. Importantly, parents, teachers, and other educational professionals may equip students with coping strategies to tackle anxiety. This would allow significant adults to promote students’ psychological well-being and ultimately their academic motivation.

Second, teachers should be mindful of their role as key sources of social support for students during the COVID-19. For example, even through remote means, teachers can establish a structured, collaborative, and autonomy-supportive classroom environment, nurturing students’ basic psychological needs and academic motivation (Deci and Ryan, 1985; Ryan and Deci, 2000, 2020).

## Conclusion

Stemming from parents’ perceptions, our study contributed to further unveil the impact of COVID-19 on students’ academic motivation, shedding light on the predictive role of students’ anxiety and teachers’ social support. Studying predictors of academic motivation is essential to understand which factors might facilitate or undermine students’ trajectories in school, especially during an ongoing pandemic. In this respect, our study highlighted the potential negative role played by COVID-19-related anxiety, that may in turn hamper academic motivation. In addition, this study underlined the potential positive role teachers can have during this pandemic as highly significant sources of social support for students in basic education.

## Data Availability Statement

The raw data supporting the conclusions of this article will be made available by the authors, without undue reservation.

## Ethics Statement

This study was approved by the Ethical Committee for the Psychological Research of the University of Padova (authorization number: 3530). The patients/participants provided their written informed consent to participate in this study.

## Author Contributions

AC and NC performed conceptualization, methodology, writing original draft, review and editing, and project administration. SZ performed conceptualization, methodology, and review and editing. JD performed data analysis, writing original draft, review and editing. All authors contributed to the article and approved the submitted version.

## Conflict of Interest

The authors declare that the research was conducted in the absence of any commercial or financial relationships that could be construed as a potential conflict of interest.
